# Patients’ experiences and perspectives of patient-reported outcome measures in clinical care: A systematic review and qualitative meta-synthesis

**DOI:** 10.1371/journal.pone.0267030

**Published:** 2022-04-21

**Authors:** Liam Carfora, Ciara M. Foley, Phillip Hagi-Diakou, Phillip J. Lesty, Marianne L. Sandstrom, Imogen Ramsey, Saravana Kumar

**Affiliations:** 1 UniSA Allied Health and Human Performance, University of South Australia, Adelaide, South Australia, Australia; 2 Rosemary Bryant AO Research Centre, UniSA Clinical & Health Sciences, University of South Australia, Adelaide, South Australia, Australia; University of Pennsylvania, UNITED STATES

## Abstract

**Background:**

Patient-reported outcome measures (PROMs) or patient-reported outcomes (PROs) are used by clinicians in everyday clinical practice to assess patients’ perceptions of their own health and the healthcare they receive. By providing insight into how illness and interventions impact on patients’ lives, they can help to bridge the gap between clinicians’ expectations and what matters most to the patient. Given increasing focus on patient-centred care, the objective of this meta-synthesis was to summarise the qualitative evidence regarding patients’ perspectives and experiences of the use of PROMs in clinical care.

**Methods:**

A systematic search of the following databases was undertaken in August 2020: Medline, EMBASE, EMCARE, PsychINFO, Scopus and the Cochrane Library. This review was conducted and reported in line with the Preferred Reporting Items for Systematic Reviews and Meta-Analyses (PRISMA) statement. Methodological quality of the included studies was assessed using the Critical Appraisal Skills Programme checklist for qualitative research (CASP). A meta-ethnographic approach was used for data extraction and meta-synthesis of findings (PROSPERO registration: CRD42020202506).

**Results:**

Fourteen studies from a range of countries with differing qualitative research methodologies were identified. Three themes were identified, namely ‘patient preferences regarding PROMs’, ‘patient perceived benefits’ and ‘barriers to patient engagement with PROMs’. The perspectives of patients suggested they preferred PROMs that were simple and relevant to their conditions and found benefits in the way they facilitated self-reflection and effective communication with their clinicians. Patients, however, questioned the relevance of some individual questions and purpose.

**Conclusion:**

PROMs can be a useful tool in the clinical setting by enabling individualisation and patient centred care. This meta-synthesis provides insights into what patients find beneficial as well as barriers to their engagement, highlighting the importance of educating patients about PROMs.

## Introduction

In response to growing recognition of the importance of patient-centred care, the last 30 years have seen a proliferation in the number of tools designed to assess patients’ perceptions of their own health and the healthcare they receive. A patient-reported outcome (PRO) is “any report of the status of a patient’s health condition that comes directly from the patient, without interpretation of the patient’s response by a clinician or anyone else [1].” Patient-reported outcome measures (PROMs)–the instruments used to assess PROs–may capture the impacts of a health condition or its treatment on quality of life, functional status, and symptoms, as well as health-related behaviours and patient experiences of and satisfaction with healthcare [2]. PROMs are typically structured and validated questionnaires that yield quantitative data, allowing comparison across healthcare providers, settings, and patient groups [2]. By providing insight into how illness and interventions impact on patients’ lives, PROMs can serve as valuable tools for improving the quality of healthcare.

The various applications of PRO data include enhancing clinical interactions and decision-making at the individual level; demonstrating the risks, benefits, and safety of interventions as well as variations in outcomes, costs, and processes at a service level; and highlighting trends and disparities at a population level [3]. Although many PROMs were initially developed for use in clinical research [4], with the wider adoption of electronic medical records, it has become feasible to collect PRO data routinely in practice and use these data in real time to support the provision of patient-centred care [5]. PROMs are used in individual patient care as screening tools (e.g., for psychological distress), for clinical monitoring of symptoms and treatment response over time, and to inform patient self-management and care planning [6]. The shift towards using PROMs in this context is thought to have been prompted by the significant body of evidence indicating that about half of the symptoms experienced by patients during treatment are missed during consultations [7]. This may result in patient suffering due to poor treatment control, missed treatments, emergency department visits, and hospitalisations [4].

Drawing definitive conclusions about the impact of using individual-level PRO data in clinical practice has been difficult due to the heterogeneity of interventions, outcomes, and indicators [8]. Systematic reviews examining the impact of routine PROM assessment in healthcare have identified strong evidence that PROs improve patient satisfaction and patient-provider communication [9], and increase the frequency of discussions about patient outcomes [10]. They have also found associations with enhanced treatment response monitoring, symptom control, and detection of unrecognised problems, although the effects on patient outcomes and supportive care needs were equivocal [8–12]. Some studies have found associated clinical benefits of PRO assessment, including lengthened survival, improved HRQOL, and reduced emergency department use [5, 13], but this evidence is still emerging [14].

At the same time, numerous barriers to the effective use of PROMs in practice have been identified [15]. Regarding the characteristics of PROMs, barriers commonly reported by clinicians include concerns about their validity and reliability, perceptions about the value of PROMs (e.g., provision of clinically valuable information) and the design process (e.g., decisions about the choice of a PROM, and the processes for PROMs data gathering, management, utilisation and interpretation), and the perceived difficulty and cost of implementing them [8, 11, 15–17]. Clinician-reported barriers pertaining to patient needs include the perceived relevance of PROMs, and concerns that they may add undue burden, cause distress, or detrimentally impact on patient care [8, 11, 15–17]. There are also technical and administrative barriers to the successful implementation and translation of PRO monitoring into practice; for example, where there is no systematic approach for using PRO data to inform patients’ treatment plans, or where PRO data cannot be easily integrated into electronic patient records [18]. It has been argued that this is because more attention has been given to the psychometric properties of PROMs than to how PRO data will improve the quality of patient care [6].

Qualitative research is vital for understanding the mechanisms and contextual factors that determine whether interventions are effective in practice and developing theoretical models to guide successful implementation [8]. Two reviews of qualitative research regarding health professionals’ perceptions of PROMs have been published [8, 19] but synthesis of the qualitative evidence regarding patients’ perspectives and experiences of PROMs has not been conducted. To bridge this gap, this systematic review and meta-synthesis aimed to summarise the qualitative evidence regarding patients’ perspectives and experiences of the use of PROMs in routine care.

## Methods

### Search protocol and registration

The protocol for this systematic review was registered with the international prospective register of systematic reviews–PROSPERO (Registration # CRD42020202506).

### Inclusion criteria

This review included qualitative research that explored experiences and perspectives of patients regarding PROMs use in healthcare, and which used thick descriptions to describe these findings. The use of thick descriptions meant authors of the qualitative research were able to provide detailed and direct quotes about the findings rather than mere descriptive summaries, or mere quantification of qualitative data. Research, which was focused on clinicians’ experiences of PROMs solely, and was not exclusively underpinned by qualitative research methodologies/methods were also excluded. All adult (i.e., over the age of 18) patient populations (i.e., any health condition or setting) were of interest. The review was limited to studies published in English.

### Search strategy and study selection

An initial search was conducted in MEDLINE (PubMed), and then translated for and undertaken across all other included databases: Embase, Emcare, PsychINFO, Scopus, and the Cochrane Library (LC & PL). The databases were searched between 10^th^ and 28^th^ August 2020 with English language limit, but no restrictions placed on date of publication. To minimise publication bias, targeted keyword searches of grey literature sources (such as Google and organisational websites) were conducted, in conjunction with hand searching of the reference lists of included studies (pearling) (LC & PL). The search combined MeSH terms and key descriptors of 1) patients, 2) experiences and perspectives, 3) PROMs, and 4) qualitative research. The search terms and parameters are provided in Table 1. The search results were exported into Endnote^™^ where the hits from databases and grey literature were merged. These results were then transferred into Covidence^™^ (Covidence.org), which is a data management software used for systematic reviews. Following removal of duplicates, two independent reviewers (LC & PL) screened first by title and abstract, followed by full text screening. Any disagreements were resolved through discussion between the reviewers.

**Table 1:** Key concepts and search terms.

**Population**	Patients/ Patient* OR Client?
**Intervention**	Patient Reported Outcome Measures/
	Patient?Reported Outcome Measure? OR Patient Reported Outcome* OR Patient Reported Outcome Measure* OR Electronic Patient?Reported Outcome Measure? OR “ePROM” OR “e-PROM” OR “PRO” OR “PROM” OR “PROMs” OR Self?reported outcome? OR Self?reported patient outcome?
**Context**	Qualitative Research/
	Interview/
	Focus Groups/
	Qualitative Study or or Health?related quality of life or HRQOL Interview? or Focus Group? or Qualitative Research
**Outcomes**	Attitude/
	Perception/
	Patient Satisfaction/
	Experience? OR Opinion* OR View? OR Perspective? OR Satisfaction OR OR Perception? OR Attitude? OR Belief?
**Limits**	English Language

**Key**: / = MeSH heading; * = truncation; ? = Wildcard; ‘OR’ = Booleans in between keywords; ‘AND’ used to combine each row of modified PICO.

### Critical appraisal

The methodological quality of the included studies was assessed using the Critical Appraisal Skills Programme checklist for qualitative research (CASP) [20], which is freely available and widely used to appraise qualitative research. Appraisal of validity was based on declaration of study aims and whether the chosen qualitative methodology was appropriate to address those aims [20]. Appraisal of bias was based on whether the relationship between the researcher and participants was considered [20]. Appraisal of ethical considerations was based on whether ethics committee approval was granted, and informed consent obtained [20]. Appraisal of data collection was based on whether data collection methods were appropriate and justified, and if the form of data was clear [20]. Appraisal of data analysis was based on clarity, rigour, and justification of the analytical approach (e.g., thematic analysis), whether biases were examined, and whether sufficient data were presented to support findings [20]. Appraisal of transferability was based on the applicability of the research to other contexts and whether adequate detail was given about the sample [20]. Studies were not excluded based on their methodological quality. However, this information was used to report, analyse and discuss the overall review findings. To ensure consistency of the critical appraisal process, independent appraisals of the studies were completed by all members of the review team (LC, CF, PH, PL & MS) and compared. Any disagreements were resolved through discussion between the review team.

### Data extraction and synthesis

A meta-ethnographic approach [21] was used for data extraction and meta-synthesis of findings from the studies deemed eligible for inclusion. This approach has previously been used in health research, especially when exploring patient experiences of their own health and healthcare they received. Therefore, given that this systematic review aimed to summarise patients’ perspectives and experiences of the use of PROMs in routine care, this approcah was an ideal choice. Meta-ethnography is therefore well-established [22], rigorous, and underpinned by three stages of analysis [23] For the first stage of analysis, one reviewer (MS) read the studies multiple times and extracted study characteristics, key findings, and quotes from participants from the included studies into a predefined template on Microsoft Word. This was particularly useful in becoming familiar with, and understanding, the content of the included studies and starting to recognise emerging themes. During the second stage of analysis, known as reciprocal translation, participant quotes from the included studies were grouped into concepts [23]. This was informed by a pragmatic (initial grouping of concepts were refined over time with consultations between reviewers) as well as a methodological (trying to maintain and staying close to the original data) approach. The third and final stage involved regular and systematic comparison and interpretation of the data, and synthesis into broad themes. As there is no one standard way to do this, this stage was informed by a previous systematic review which also used a meta-ethnographic approach [22].

To enhance rigour and minimise biases, the entire process was overseen and reviewed by another member of the review team (SK).

## Results

### Search outcomes

**Fig 1:**
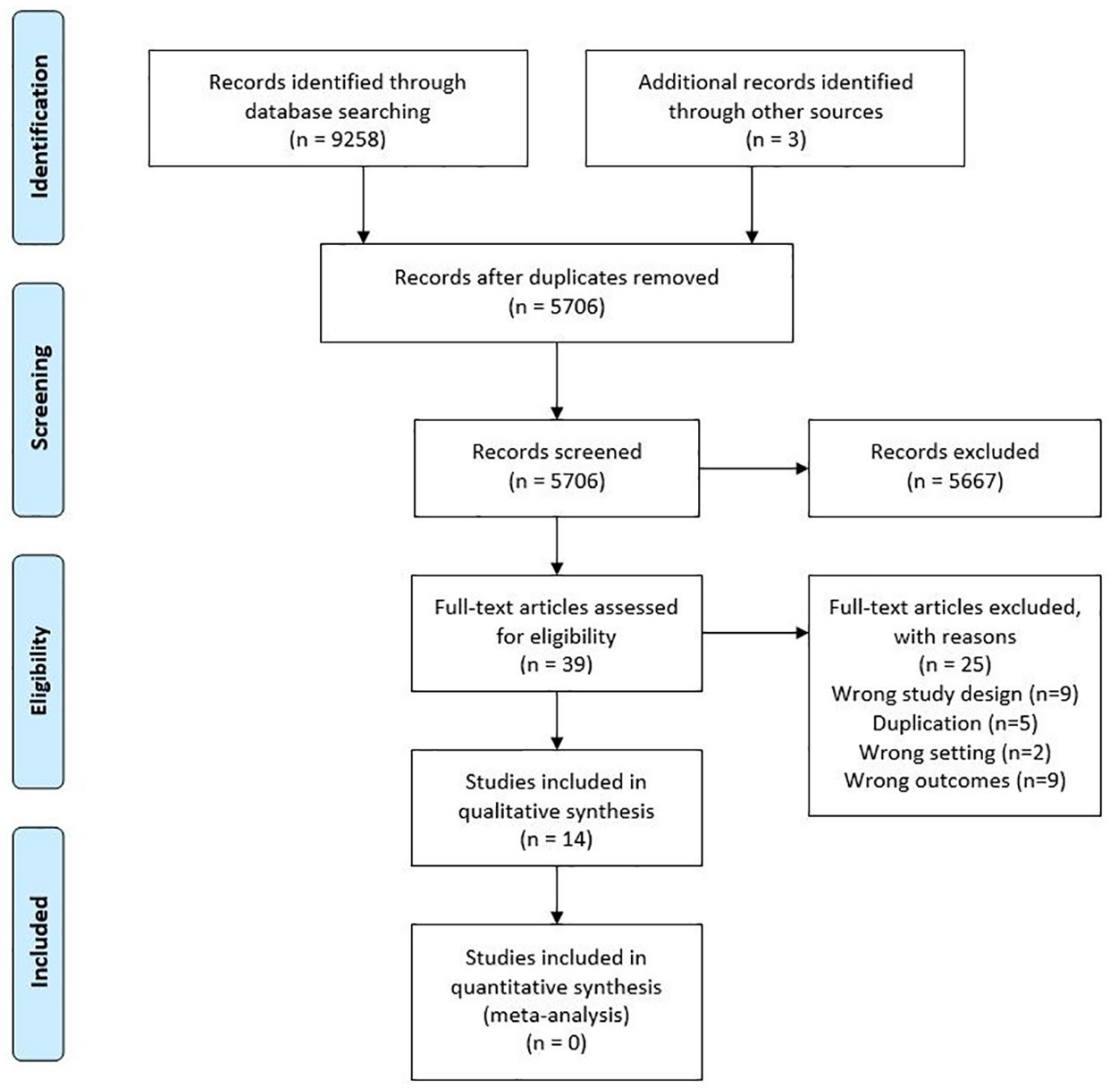
PRISMA flow chart.

The literature search yielded a total of 9261 articles, including 9258 records from database searches and a further three studies were identified through pearling. After the removal of duplicates, the titles and abstracts of 5706 articles were screened against the inclusion criteria in Covidence^™^. The title and abstract screen identified 39 potentially relevant studies for full text review. Of these, 25 studies were excluded for reasons documented in Fig 1. The remaining 14 studies were deemed eligible and included in the review [24–37]. The selection process is documented in Fig 1.

### Characteristics of included studies

Four of the studies originated in the United States of America [26, 28–30], four in Demark [31–34], three in the United Kingdom [24, 25, 35], two in The Netherlands [27, 36] and one in Sweden [37]. While all the studies were qualitative, three [26, 30, 34] reported the qualitative findings of mixed-methods research projects. Six involved thematic analysis (inductive or theoretical) [24, 25, 27, 28, 30, 36], three reported using interpretive description [31, 32, 34], and the remaining studies reported conducting content analysis [33], framework analysis [26], systematic text condensation [37], constant comparison [35] and Delphi consensus methods [29]. All of the studies involved face-to-face and/or phone semi-structured interviews, with the exception of four studies [28, 29, 33, 37] that collected patient experiences and perspectives via face-to-face focus groups.

The majority of the studies involved purposive sampling of adult male and female outpatients living with a chronic condition (including chronic kidney disease, inflammatory arthritis, depression, chronic haematological disease, Parkinson’s disease, epilepsy and chronic musculoskeletal presentations), with sample sizes ranging from 9 to 32 individuals. One study included patients with acute musculoskeletal presentations [27]. Two studies did not report participant gender [25, 26], and one also did not report participant age [26]. Two studies [24, 25] did not explicitly state that their sample comprised outpatients.

All of the studies collected participant experiences and perspectives of PROMs (digital and/or hardcopy, condition-specific and/or generic) use. The PROMs either had been used in the past and/or were currently in use, or had been implemented as part of the study, with the exception of two studies [24, 28] that involved conceptual PROMs (i.e., the idea of implementing PROMs). The characteristics of individual studies are presented in Table 2.

**Table 2:** Characteristics of the studies included in meta-synthesis.

Author(s) & Year	Country	Focus of Research	Methodology	Sample & Sample Characteristics (n)	PROM(s)
Aiyegbusi et al. 2019 [24]	United Kingdom (England)	General opinions, practical considerations, concerns, and perceived potential barriers and enablers associated with the use of a renal electronic patient-reported outcome measure (ePROM) system	Qualitative Thematic Analysis Semi-Structured Interview	Sample size: n = 12 Sampling approach: Purposive Age: aged ≥ 50yrs (11) Gender: Male (7), Female (5) Condition: Stage 4 & 5 chronic kidney disease (CKD) (Pre-Dialysis)	Conceptual ePROM, wrt Kidney Disease Quality of Life-36 (KDQOL-36), Integrated Patient Outcome Scale Renal (IPOS-Renal)
Aiyegbusi et al. 2020 [25]	United Kingdom (England)	Perspectives on potential benefits, practical considerations for implementation, and barriers and enablers of implementation associated with the use of PROMs/ePROMs in the routine management of rare diseases	Qualitative Inductive Thematic Analysis Semi-Structured Interview	Sample size: n = 9 Sampling approach: Purposive Age: 16-25yrs (renal transplant recipients); 18-80yrs (primary sclerosing cholangitis) Gender: Not reported Conditions: Renal transplant (5); primary sclerosing cholangitis (4)	Chronic Liver Disease Questionnaire (CLDQ), Short Form 12 (SF12), Paediatric Quality of Life Inventory Transplant Module (PedsQL-TM) and EuroQoL-5 Dimensions (EQ-5D) = 5x, Pts with RTR = PedQL-TM & EQ-5D, Pts with PSC = CLDQ & SF12
Bartlett et al. 2020 [26]	United States of America	Perspectives on the influence of clinical interactions, the value of patient-reported outcomes (PROs), and confidence in treatment decisions associated with the use of a comprehensive set of PROs as part of routine rheumatology visits	Mixed-Methods Framework Analysis Semi-Structured Interview	Sample sizes: n = 9 at midway; n = 15 at end Sampling approach: Purposive Gender: Not reported Condition: Rheumatoid Arthritis	Patient-Reported Outcomes Measurement Information, System (PROMIS, regarding pain interference, physical function, fatigue, participation in social roles and activities, satisfaction with social roles, sleep disturbance, sleep interference, anxiety, depression and anger) in general
Damman et al. 2019 [36]	The Netherlands	Current experience, comprehension of various PROMs data, explicit information needs, and preferences of receiving PROMs data associated with the use of PROMs during routine medical consultations	Qualitative Inductive Thematic Analysis Semi-Structured Interview	Sample size: n = 13 Sampling approach: F2F Recruitment, Ads OR Invitation (Neurologist) Age: <65 to ≥75yrs Gender: Male (4); Female (9) Condition: Parkinson’s Disease	PROMs in general
Dowrick et al. 2009 [35]	United Kingdom (England)	Perspectives on utility, validity, importance, and potential manipulation associated with the use of severity questionnaires for depression	Qualitative Constant Comparison Semi-Structured Interview	Sample size: n = 24 Sampling approach: Purposive, Ads OR Invitation (GP) Age: 20-77yrs Gender: Male (9); Female (15) Condition: Depression	Depression severity questionnaires in general
Meerhoff et al. 2019 [27]	The Netherlands	Perspectives on practicality, clinical interactions for decision-making, and information sharing associated with the use of PROMs in primary care physiotherapy practice	Qualitative Theoretical Thematic Analysis Semi-Structured Interview	Sample size: n = 21 Sampling approach: Invitation (Physiotherapist) Age: 24-76yrs Gender: Male (6); Female (15) Conditions: Musculoskeletal health problems (Acute (14); Chronic (7))	PROMs in general
Trillingsgaard et al. 2016 [31]	Denmark	Perspectives on the influence of patient-clinician interaction during the consultation associated with the use of a web-based PRO system in an outpatient clinic	Qualitative Interpretive Description Semi-Structured Interview	Sample size: n = 12 Sampling approach: Referral (Nephrologist) & Consecutively by convenience Age: 36-81yrs Gender: Male (9); Female (3) Condition: CKD	AmbuFlex/Pre-Dialysis (PRO-based self-management)
Mejdahl et al. 2018 [32]	Denmark	Perspectives on supporting and inhibiting mechanisms associated with the use of PRO-based follow-up in outpatient clinics	Qualitative Interpretive Description Semi-Structured Interview	Sample size: n = 29 Sampling approach: Purposive & Theoretical Age: 20 to >65yrs Gender: Male (14); Female (15) Condition: Epilepsy	AmbuFlex/Epilepsy (PRO-based follow-up)
Navarro-Millan et al. 2019 [28]	United States of America	Attitudes, beliefs, and perceived barriers and facilitators associated with electronic communication and PRO data collection	Qualitative Thematic Analysis Focus Group	Sample size: n = 31 Sampling approach: Referral (Provider) OR Ads Age: 25-84yrs Gender: 6% Male; 94% Female Condition: Rheumatoid Arthritis	Conceptual ePROM
Philpot et al. 2017 [29]	United States of America	Commonly reported barriers and benefits associated with PRO implementation	Qualitative Delphi Technique Focus Group	Sample size: n = 10 Sampling approach: Recruited Age: 35-74yrs Gender: Male (5); Female (5) Conditions: >1x Chronic Health Condition PLUS >1x Outpatient Care Visit 12/12	PROs in general such as Short Form 36 (SF 36) and Chronic Obstructive Pulmonary Disease Assessment Test
Primdhal et al. 2020 [33]	Denmark	Experience and perceived potential improvements associated with the collection and use of PROs as part of routine care	Qualitative Content Analysis Focus Group	Sample size: n = 32 Sampling approach: Invitation (online) & Purposive Age: 32-80yrs Gender: Male (11); Female (21) Conditions: Rheumatoid Arthritis (21); Psoriatic Arthritis (6); Axial Spondyloarthropathy (5) PLUS Diagnosis >12/12	DANBIO PROMs (specifically Health Assessment Questionnaire (HAQ), ~Multi-Dimensional Health Assessment Questionnaire (MD-HAQ) and Visual Analogue Scale (VAS), + Bath Ankylosing Spondylitis Disease Activity Index (BASDAI) and Functional Index (BASFI), Pts with PsA &/OR axSpA) in general
Talib et al. 2018 [30]	United States of America	Perceived benefits and limitations associated with the use of symptom-based PROs in primary care.	Mixed-Methods Thematic Analysis Semi-Structured Interview	Sample size: n = 23 Sampling approach: Invitation (from larger RCT) Age: 24-77yrs Gender: Male (12); Female (11) Condition: >1x sleep, pain, anxiety, depression, and low energy/fatigue symptoms	Patient-Reported Outcome Measurement Information System (PROMIS, regarding fatigue, pain, sleep, anxiety and depression) in general
Threstrup Hansen et al. 2019 [34]	Denmark	Experience with participating in a randomised PROM intervention study, including invitation to participate, completion of the PROM questionnaires, and subsequent visits to the outpatient clinic	Mixed-Methods Interpretive Description Semi-Structured Interview	Sample size: n = 16 Sampling approach: Purposive (from larger RCT) Age: 68-86yrs Gender: Male (10); Female (6) Condition: Chronic Haematological Disease	The European Organisation for Research and Treatment of Cancer (EORTC) Quality of Life Questionnaire (QLQ-C30) and the Outcomes and Experiences Questionnaire (OEQ)
Wikberg et al. 2016 [37]	Sweden	Experience, and perceived benefits and limitations associated with the use of Montgomery-Åsberg Depression Self-Assessment Scale (MADRS-S) in primary care consultations with GPs	Qualitative Systematic Text Condensation Focus Group	Sample size: n = 9 Sampling approach: Invitation (from larger RCT) Age: 18 to >45yrs Gender: Male (1); Female (8) Condition: Mild-Moderate Depression	MADRS-S

### Methodological quality

All of the studies were evaluated as having a clear statement of the aims of the research, an appropriate qualitative methodology, data collection that addressed the research question(s), sufficiently rigorous data analysis, and a clear statement of findings. Two studies [24, 25] did not explicitly state their research design. Three studies [27, 31, 36] had recruitment strategies that involved treating therapists identifying and directly inviting patients to participate, which was considered to be inappropriate. None of the articles included researcher declarations, but three [27, 36, 37] included statements referring to management of the relationship between researchers and participants. The critical appraisal did not result in the exclusion of any studies or influence the meta-synthesis. The results of the critical appraisal are summarised in Table 3.

**Table 3:** Methodological quality of included studies.

		Publications													
CASP Qualitative Research Questions		Aiyegbusi et al. 2019 [24]	Aiyegbusi et al. 2020 [25]	Bartlett et al. 2020 [26]	Damman et al. 2019 [36]	Dowrick et al. 2009 [35]	Meerhoff et al., 2019[27]	Trillingsgaard et al. 2016 [31]	Mejdahl et al.,2018 [32]	Navarro-Millan et al., 2019[28]	Philpot et al. 2017 [29]	Primdahl et al. 2020 [33]	Talib et al. 2018 [30]	Threstrup Hansen et al., 2019 [34]	Wikbeg et al. 2016 [37]
1.	Was there a clear statement of the aims of the research?	Yes	Yes	Yes	Yes	Yes	Yes	Yes	Yes	Yes	Yes	Yes	Yes	Yes	Yes
2.	Is a qualitative methodology appropriate?	Yes	Yes	Yes	Yes	Yes	Yes	Yes	Yes	Yes	Yes	Yes	Yes	Yes	Yes
3.	Was the research design appropriate to address the aims of the research?	Can’t Tell	Can’t Tell	Yes	Yes	Yes	Yes	Yes	Yes	Yes	Yes	Yes	Yes	Yes	Yes
4.	Was the recruitment strategy appropriate to the aims of the research?	Yes	Yes	Yes	No	Yes	No	No	Yes	Yes	Yes	Yes	Yes	Yes	Yes
5.	Was the data collected in a way that addressed the research issue?	Yes	Yes	Yes	Yes	Yes	Yes	Yes	Yes	Yes	Yes	Yes	Yes	Yes	Yes
6.	Has the relationship between researcher and participants been adequately considered?	Can’t Tell	Can’t Tell	Can’t Tell	Yes	Can’t tell	Yes	Can’t Tell	Can’t Tell	Can’t tell	Can’t Tell	Can’t tell	Can’t Tell	Can’t Tell	Yes
7.	Have ethical issues been taken into consideration?	Yes	Yes	Yes	Yes	Yes	Yes	Yes	Yes	Yes	Yes	Yes	Yes	Yes	Yes
8.	Was the data analysis sufficiently rigorous?	Yes	Yes	Yes	Yes	Yes	Yes	Yes	Yes	Yes	Yes	Yes	Yes	Yes	Yes
9.	Is there a clear statement of findings?	Yes	Yes	Yes	Yes	Yes	Yes	Yes	Yes	Yes	Yes	Yes	Yes	Yes	Yes
10.	How valuable is the research?	Valuable	Valuable	Valuable	Valuable	Valuable	Valuable	Valuable	Valuable	Valuable	Valuable	Valuable	Valuable	Valuable	Valuable

### Findings

The meta-synthesis of 14 studies resulted in the identification of three overarching themes: patient preferences regarding PROMs, patient-perceived benefits of PROMs, and barriers to patient engagement with PROMs.

#### Theme 1—Patient preferences regarding PROMs

The patient preferences regarding PROMs identified cover a range of topics including type and nature of questions, formatting, medium (electronic versus hardcopy), environment (clinic versus home), timing, and other applications.

Three studies found that patients preferred PROMs with relevant questions [25, 27, 29] and two studies [25, 29] reported that patients preferred condition-specific PROMs over generic PROMs.

“*Yes*, *the patient reported outcome measures (PROMs) are applicable to my health problem*, *and therefore*, *it’s relevant to answer such questions*.”

[27]

Some patients suggested PROM questions need to be more nuanced if they are to accurately capture the nature of their fluctuating symptoms [27, 30, 33], with comment boxes “to specify something” [33] and/or the option to “tick a box” [33] to flag priority discussion points.

“*The disadvantage of such questionnaires is that they measure a specific moment in time*. *That is difficult since my health problems differ each day*. *Every once in a while when I fill in the questionnaire on a relatively good day I wonder if my physiotherapist gets a representative picture*.”

[27]

In addition, patients favoured PROMs formatted with fewer questions, consistent instructions, and simple, intuitive graphics [29, 30, 33, 34, 36].

“*I think there are so many questions and different instructions*. *Sometimes I am told to mark with a circle [around the response categories]*, *and sometimes I am told to mark with a cross*. *[…]*”

[34]

Patients had mixed preferences regarding completing electronic versus paper PROMs [25, 27, 30, 33, 34]. These mixed preferences regarding medium were also reported in the conceptual studies [24, 28]. Patients who preferred electronic PROMs cited improvements to completion time, data accuracy, data management “*because paper has a way of getting lost*” [30], health system efficiency, and environmental considerations as key reasons [25, 30, 33, 34].

“*I don’t mind paper*, *but I would prefer electronically*. *First of all*, *it’s environmentally friendly and—it’s also much better to keep the record electronically*, *isn’t it*? *less hassle*, *it’s much quicker*, *it’s more efficient*, *more accurate…*”

[25]

Patients who favoured paper PROMS identified a lack of affinity for computers plus concerns about data security and hygiene as explanations [25, 27, 30].

“*Well*, *we are asked to complete the questionnaire using a computer and that is a little difficult for me because I am a little older and… well*, *it is only recently that we have had a computer*.”

[27]

Patients had mixed preferences regarding completing PROMs in the clinic versus at home [25, 27, 33]. Patients who preferred completing PROMs in the clinic reported that it gave them something to do “*while I’m waiting for the Doctor*” [25]. Patients who favoured completing PROMs at home identified increased privacy “*to get the most honesty out of people*” [25], convenience, and the preservation of consultation time as benefits [25, 27, 33].

“*I think it is more convenient to fill in the questionnaire when you are at home*, *at a time that it suits yourself*. *At least then it will not go at the expense of your consultation time… […]*”

[27]

Aiyegbusi et al., [25] reported patients had mixed preferences regarding the frequency at which PROMS should be completed (ranging from 3–12 months) but that, in general, they thought PROMs should coincide with consultations because “*I come in every year anyway*” [25]. Mixed preferences regarding timing were also reported in the conceptual study by Aiyegbusi et al., [24]. Some patients suggested completing PROMs between consultations “*would help spot trends that might come up or missed symptoms*” [26]. One patient recommended PROMs closely follow any new diagnosis because “*it is very important to get all the information immediately*” [36].

Aiyegbusi et al., [25] and Meerhoff et al., [27] reported patients were amenable to sharing their PROM data for patient care, clinician training “*to learn from each other*” [27] and/or research purposes. This amenable perspective towards other applications was also reported in the conceptual study by Aiyegbusi et al., [24]. However, Meerhoff et al., [27] reported the majority of patients were reluctant to share their PROM data with insurance companies “*because insurance companies are commercial entities*, *for whom obtaining profit is a central theme*” [27].

“*I don’t really mind*. *I mean*, *if it helps in research or anything*, *then*, *you know*, *I’m all for it*.”

[25]

#### Theme 2—Patient-perceived benefits of PROMs

The identified patient-perceived benefits of PROM applied to patients, clinicians and the patient-clinician relationship, with key concepts including self-reflection, facilitation and communication.

Patients reported PROMs caused them “*to stop and think*” [30], which prompted self-reflection on their condition [26, 27, 30, 35]. However, Mejdahl et al., [32] reported some patients were distressed by self-reflection.

“*It made me think more about what actually was happening*. *I knew there was sleep disruption; it made me analyze it more*… *helped me identify problems and think more deeply about them*”

[26]

PROMs increased patients’ understanding of their condition “*because it organizes how you really feel*” [26, 27, 31, 32, 37]. This increased understanding through self-reflection was also reported as a patient benefit in the conceptual study by Aiyegbusi et al., [24].

“*Before I start to fill it in*, *I stop and think carefully about why the questions are there in the first place*. *They must be linked to the epilepsy*. *[…] And that makes you aware of symptoms that you must be attentive to*.”

[32]

Mejdahl et al., [32] reported that some patients were empowered by PROMs.

“*I actually think that just filling in the questionnaire and just by ticking those boxes made me more conscious*, *and then I said to myself* “*okay now I need to take on responsibility*, *because it is my life*.”

[32]

Patients reported PROMs facilitated their planning in preparation for consultations because “*it kind of jogs your memory*” [25, 30, 34].

“*Sometimes we [participant and his wife] talk about the questions and what did I answer last time… or sometimes this reminds me something that I should ask the nurse*.”

[34]

PROMs increased patients’ sense of their “*being taken more seriously*” [32, 35, 37].

“*At least the questionnaire is more profound than the usual how-are-you-questions*. *It seems as if they take you a bit more seriously now than they did before*.”

[32]

Patients reported that PROMs facilitate increased clinician understanding of their condition by providing them with a “*fuller (…) more accurate picture*” [25, 30, 35].

“*[…] (It) should be mandatory*. *That way the doctors get a feel of how you are feeling or how you think you are feeling*.”

[30]

PROMs facilitated clinician planning, assessment, diagnosis, treatment and monitoring [26, 27, 30, 35, 37], which improved an individualised approach to patients [27, 35, 37] and increased efficiency for patient visits [29]. This facilitation of processes was also reported as a patient benefit in the conceptual study by Aiyegbusi et al., [24].

“*Obviously the benefit of using PROMs is that PTs can prepare themselves for my visit*. *Using the PROM results*, *your PT (physical therapist) can analyze what might trigger the health problem and think about the intervention they might use*. *At a later phase*, *when the PROMs are completed again*, *they could analyze the progression did the pain decrease or is it completely resolved*?“

[27]

“*[…] You can of course certainly end up with more accurate treatment by filling in this kind of questionnaire*.”

[37]

“*These questions take out the fluff and other things that the doctors talk about and focuses them on the issue*. *I think it helps the doctor*”

[29]

Patients reported PROMs improved patient-clinician communication by providing an “icebreaker” [29], structure and condition-specific vocabulary, highlighting priorities plus opening-up the discussion to broader issues [29, 32], which increases mutual understanding [37] and enables shared decision-making [26]. This improved communication was also reported as a patient-clinician relationship benefit in the conceptual studies by Aiyegbusi et al., [24] and Navarro-Millan et al., [28].

“*Well*, *I think it is very nice*. *The questions are much more everyday questions*. *That makes it much easier to explain and describe how your epilepsy actually is*. *Because you try and you try to explain how it is and how it feels to your close ones and to the doctors*, *but it is so hard to explain in a way that normal people can imagine how your body experiences it*.”

[32]

“*It was extraordinarily helpful when you sit down with your doctor and he says*, “*This is what I see*…” *and I say*, “*Well this is what I see*…”. *And we talk about how we can improve those areas that I would like to see improved*, *or maybe manage better those areas that may not be able to be improved*”

[26]

Patients living with depression and/or anxiety appreciated that PROMs enabled them to communicate difficult information impersonally [30, 31, 35]. However, Dowrick et al., [35] and Primdahl et al., [33] reported some patients manipulated PROM data to minimise stigma and/or influence treatment. This PROM susceptibility to manipulation was also reported in the conceptual study by Aiyegbusi et al., [24].

“*[…] Sometimes I don’t want to tell people how I’m feeling*. *You know*, *pride or don’t want to bring somebody else down*. *So this way it’s a more impersonal way of answering the questions because you’re just marking on paper; you’re not actually telling somebody face-to-face*.”

[30]

“*You’re more likely to lie*, *well I found I’m more likely to lie*… *Because I still find a lot of stigma attached to depression*”

[35]

#### Theme 3—Barriers to patient engagement with PROMs

The barriers to patient engagement with PROMs identified cover a range of topics including patients’ capacity to answer, question relevance and validity, patients’ understanding of purpose and inconsistent clinical utilisation.

Some patients questioned whether or not they were qualified to answer PROMs [32–34].

“*Well*, *we are not supposed to be doctors*, *we are not supposed to assess our own health*, *because there are some people who are professionally educated to do that*”

[32]

Alternately, some patients questioned the relevance of individual PROM questions [32, 34] and/or their accuracy for truly capturing patient’s symptoms and experiences [30, 33, 35]. Damman et al., [36] reported some patients thought PROMs didn’t tell them anything they didn’t already know or told them things they didn’t want to know.

“*[…] I think the questions are silly*. *Take me for an example; my spine is collapsed*, *and I have pain in my shoulders and hip*. *Then you ask if I have pain*. *I answer yes*. *Nevertheless*, *this has nothing to do with my leukemia*. *[…]*”

[34]

“*You fill out a questionnaire that says what’s your level of pain*. *You write that down but you don’t know that person’s level of pain… You could say to me*, *yes I’m in pain*, *but I don’t know your pain… Do they really know*?”

[30]

“*Why would it help me to know that my situation has greatly worsened*?”

[36]

Some patients reported that they did not know the purpose of PROMs [33, 34], with one patient commenting “*if you knew the purpose (*…*) one would take it a bit more seriously*” [33].

“*I did not know if my physician got my answers or if this study was running concurrent*. *I guess it is some huge and broad research with control groups and everything… someone must use it for something*. *[…]*”

[34]

Alternately, some patients reported that the purpose of PROMs was limited to clinician- and/or research-related applications rather than anything patient-specific [31–34].

“*Well*, *they can’t use my part in itself*. *I think it is more in general that they use it*. *To see if people who take these medicines and who have epilepsy*, *to see if they have a direction*, *I think*. *So*, *I don’t think*, *that they use exactly my questionnaire*, *except as one in many*.”

[32]

“*Actually*, *I’ve always believed that it was something that was saved in some big database or other somewhere and that there were researchers who sat and looked at it*. *I had actually never understood completely that it was something that one could use in the consultation*”

[33]

Some patients reported that PROMs were used inconsistently during consultations [31, 33, 36]. Talib et al., [30] reported that some patients identified a relationship between utilisation and their perceptions of value.

“*Well… (long break)*. *I don’t really know*. *I don’t think he (the doctor) has talked about it (long break)*. *Yes*, *maybe once when the doctor said it looked really nice but otherwise there was nothing*.”

[31]

“*In order to get them (patients) to understand the value of it*, *of filling out the information*, *the doctor needs to use it… I mean why am I gonna fill it out if they’re not gonna look at it*? *[…]*”

[30]

## Discussion

This systematic review examined the findings of qualitative studies with thick descriptions exploring patients’ experiences and perspectives of PROMs in healthcare. The meta-synthesis identified three overarching themes: patient preferences for PROMs, perceived benefits of PROMs, and perceived barriers to the use of PROMs. Regarding preferences for PROMs, relevance of content and specificity to the disease or condition were considered important, as was presentation in a simple and consistent format. However, there was variability in preferences for how, where, and how often PROMs should be administered. Patients’ perceived benefits of PROMs related to gaining a sense of empowerment through self-reflection and providing information to assist with clinical planning, assessment, diagnosis, individualised treatment, decision-making, and monitoring. Barriers to engagement with PROMs reported by patients included patients’ capacity to provide credible information, perceived relevance and utility of the questions, understanding of purpose, and concerns about how information gathered is applied clinically.

The value patients placed on the relevance and specificity of PROMs in the included studies is consistent with findings from other evidence syntheses. A scoping review [11] found that a key factor for successful implementation of PROMs was ensuring that measures addressed issues relevant to patients. Relevance and specificity were also considered key indicators of the value of PROMs by clinicians in a systematic review of qualitative evidence [19]. To ensure relevance, careful consideration should be given to instrument selection, prioritising those developed in consultation with the patient groups they represent to capture information that is most meaningful to them. The use of individualised PROMs, which allow patients to select or weight issues of most personal relevance, may be particularly useful when significant differences between patients in priorities and areas of concern are anticipated [38]. Improved precision and efficiency of PRO assessments may also be achieved by using PROMs that utilise computer adaptive testing (CAT) algorithms to select the most relevant items for the respondent. In addition to relevance, other factors that have been found to influence the implementation of PROMs include their length and complexity, the availability of translated and culturally meaningful versions, and respondents’ comfort level with technology if completing them electronically [11].

The variability evident in patients’ preferences for paper-based and online PROMs completion is consistent with findings from the realist synthesis of PROMs by Greenhalgh et al., [6], which found that patients expressed preferences for different types of PROMs and different modes of administration. As preferences may influence how individuals respond to and engage with PROMs, identifying them and adapting processes accordingly may optimise the potential benefits of completing PROM assessments. A systematic review of reviews examining factors that influence the implementation of PROMs found that a key enabler to designing the PROMs process in an organisation was ensuring adaptability to both the organisational context and to specific patients [15]. This requires awareness of patients’ unique characteristics, needs, and resources [15], to ensure that PROMs are appropriate for, and equivalently applicable to, each individual [38]. Examples of individual factors that may influence patients’ preferences for, interpretation of, and responses to PROMs include age, disability, cultural background, education, health literacy, and previous healthcare experiences [38].

A commonly perceived benefit of PROMs reported in many of the included studies was improved patient-clinician communication (e.g. [24, 26, 28, 30–33, 35]), which was seen to facilitate clinical assessment, diagnosis, and monitoring of issues [26, 27, 30, 35–37], as well as shared-decision-making [26]. This is congruent with qualitative research on clinicians’ perspectives of PROMs, which has found that they are typically used for clinical assessment purposes and to identify issues for discussion, confirm knowledge of patients’ problems, and inform patient management [8, 19]. Similarly, quantitative studies have found the use of PROMs resulted in HRQOL issues being discussed more frequently [39–44], facilitated patient-clinician communication [39, 45], and improved patient management [41, 43].

Patients’ capacity to complete PROMs has been previously identified as a barrier to the use of PROMs in cancer care [15]. However, while this meta-synthesis identified concerns about whether patients are qualified to accurately assess their own symptoms, other reasons identified in the literature include disability or difficulty reading and/or responding to questions, being too unwell, and difficulty recalling symptoms or remembering to complete PROMs [46]. These findings highlight the importance of ensuring PROMs are user-friendly and provided in conjunction with adequate support. Just as patients in the included studies expressed concerns about how PRO data is applied clinically, negative perceptions about the value and clinical usefulness of PROMs have been identified as barriers to their use by clinicians in qualitative research [19, 46]. Therefore, efforts should be made to ensure clarity regarding the purpose of PRO data collection and how data will be used, that appropriate instruments are selected, and that patients and clinicians are informed of these details [46].

A strength of this review was the inclusion of mixed-methods studies with qualitative content in addition to qualitative studies, which enabled examination of findings from study designs often employed in research on the development and piloting of PROMs that may be excluded from other qualitative syntheses. The included studies were based in a range of countries and healthcare contexts and presented the views of various patient groups involved in the use of PROMs. In many cases, studies included well-established patient measures, but also included views about PROMs as a general concept.

While the review methods followed best practice guidance in the conduct of meta-analyses (PRISMA), there are several potentially limiting factors. Language and publication bias are likely present given that the review included only studies published in English. However, there were seven studies included from non-English speaking countries, suggesting the impacts of language bias were minimised. To reduce publication bias, searches of grey literature and reference list pearling were conducted but these did not identify any sources for inclusion. Although the search strategy was comprehensive, it was not an exhaustive search of all databases and all potential sources of grey literature, thus it is possible that relevant studies may have been missed (e.g. particular focus on patient perspectives of PROMs and excluding clinician perspectives). Limitations were also present in the included evidence base. As identified through critical appraisal, some studies did not provide a clear description of the research methodology, and in some the role of the researcher was potentially unclear, resulting in trustworthiness issues.

This meta-synthesis summarises qualitative evidence about the experiences and perspectives of patients regarding the use of PROMs in healthcare. The variability in preferences expressed by patients suggests that a one-size-fits-all approach to implementing PROMs may not be adequate, which is further supported by the value patients placed on the relevance and specificity of PROM content. To optimise the relevance of PROMs, careful consideration of the patient, context, and instrument may be required. The findings also highlight the importance of having a clear purpose for collecting PRO data and understanding of how data will be applied clinically and communicating this information to patients. The benefits of PROMs perceived by patients in the included studies aligned with literature indicating that PROMs can facilitate communication, individualisation of care, identification of concerns, and shared decision making. The evidence base identified in this review highlights the need for more in-depth inquiry into the perceived value and benefits of PROMs (e.g which patient groups are most likely to benefit from PROMs, optimal means of communicating the value of PROMs to patients etc), preferences for their delivery, and barriers to their use from the perspective of patients.

## Supporting information

S1 Table PRISMA checklist. (DOC)

S1 Fig (TIF)
