# Supplementary figures and images for: Patients’ experiences and perspectives of patient-reported outcome measures in clinical care: A systematic review and qualitative meta-synthesis
Source: PLoS One. 2022 Apr 21;17(4):e0267030. doi: 10.1371/journal.pone.0267030 (PMC9022863; doi:10.1371/journal.pone.0267030)

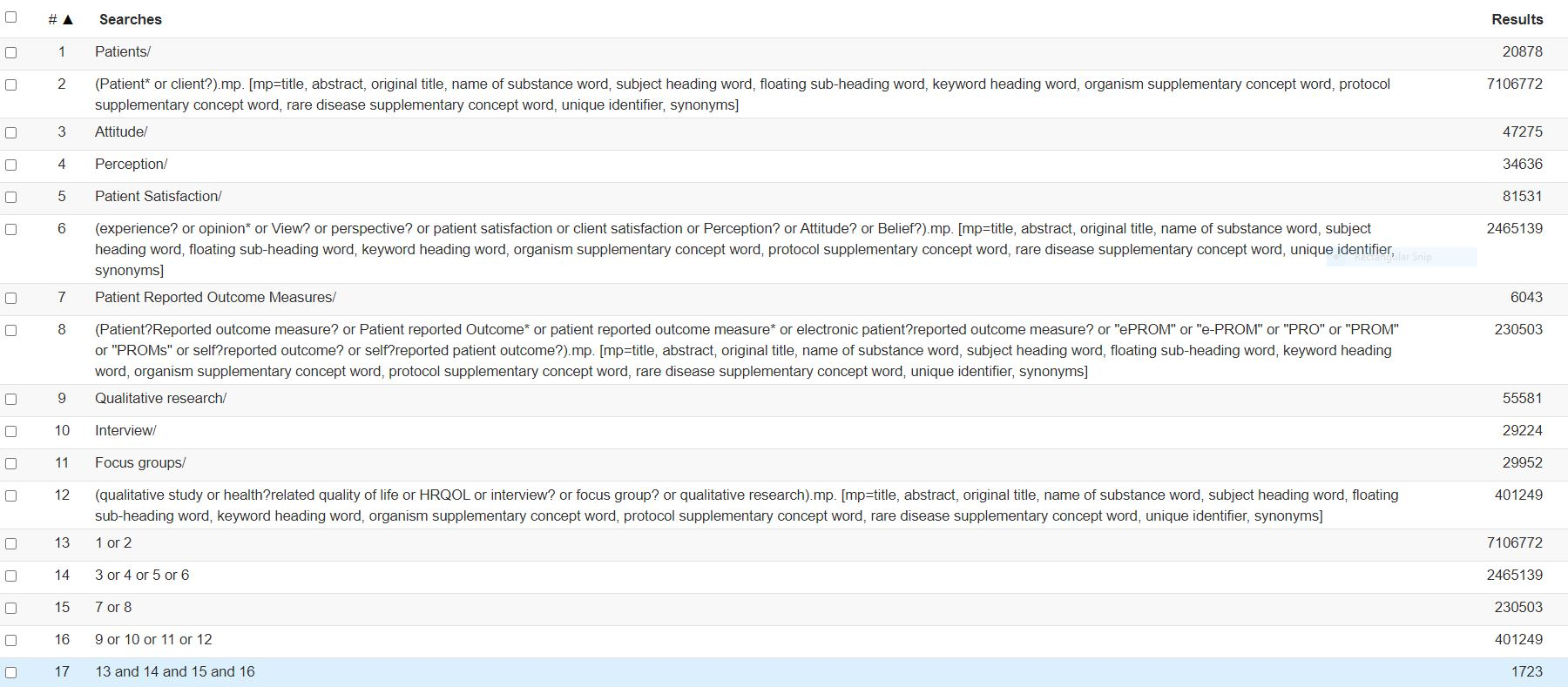

Supplement: S1 Fig — (TIF) [file pone.0267030.s002.tif]
